# A minimum of two years of undertreated primary hypothyroidism, as a result of drug-induced malabsorption of l-thyroxine, may have metabolic and cardiovascular consequences

**DOI:** 10.1016/j.jcte.2019.100189

**Published:** 2019-04-10

**Authors:** Salvatore Benvenga, Rachele Pantano, Giovanna Saraceno, Luigi Lipari, Antonio Alibrando, Santi Inferrera, Giuseppe Pantano, Giuseppe Simone, Sebastiano Tamà, Riccardo Scoglio, Maria Giovanna Ursino, Carmen Simone, Antonino Catalano, Umberto Alecci

**Affiliations:** a Dipartimento di Medicina Clinica e Sperimentale, Università di Messina, Messina, Italy; b Programma Interdipartimentale di Endocrinologia Molecolare Clinica e Salute Endocrina della Donna, AOU Policlinico G. Martino, Messina, Italy; c Istituto Auxologico Italiano, Verbania, Italy; d Medico di Medicina Generale, ASP 5, Messina, Italy; e Società Italiana di Medicina Generale, Firenze, Italy; f Biologo Nutrizionista, Endocrinologia dell’Infanzia, dell’Adolescenza e della Donna, Università di Messina, Italy

**Keywords:** Subclinical hypothyroidism, Levothyroxine malabsorption, Diabetes mellitus, Dyslipidemia, Hypertension, Cardiovascular diseases

## Abstract

**Objective:**

Cross-sectional studies have reported that TSH above or close to the upper normal limit correlates with unfavorable metabolic and cardiovascular outcomes. Certain medications impair intestinal absorption of levothyroxine (L-T4), resulting in undertreated hypothyroidism (*viz*. failure of serum TSH to reach target levels, if hypothyroidism is primary).

Further to evaluating the magnitude of sub-optimally treated primary hypothyroidism as a result of co-ingestion of those medications, we wished to ascertain whether the above complications would occur during a low number of years under polypharmacy.

**Method:**

In this retrospective study in collaboration with 8 family physicians, we enrolled adults with primary hypothyroidism under L-T4 therapy that, for 2 years minimum, was not associated with those medications (non-exposure, baseline) and that, for another 2 years minimum, it was (exposure). Outcomes were serum levels and proportions of serum TSH levels >4.12 mU/L, and proportions of complications. Complications were aggravation of pre-existing or *de novo* onset of any of metabolic syndrome, impaired fasting glycemia (IFG), diabetes mellitus, dyslipidemia, hypertension, coronary heart disease (CHD), cerebrovascular disease (CVD).

**Result:**

A total of 114 patients were enrolled. Duration of exposure to the interfering medication was 32.1 ± 6.9 months (median 31; range 24–55). Compared with non-exposure, the exposure period resulted in greater TSH levels (2.81 ± 3.62 [median 1.79] *vs* 1.27 ± 1.34 [median 0.93], P = 2.2 × 10^−20^) and proportions of values >4.12 mU/L (18.5% *vs* 4.7%, P = 1.2 × 10^−7^). Seventy-six patients (67%) had complications, whose rates of TSH >4.12 mU/L were greater than in the 36 complication-free patients (22% *vs* 11%, P = 0.018).

**Conclusion:**

During a median period of 31 months, there are relevant consequences for L-T4 treated adult hypothyroid patients resulting from hyperthyrotropinemia caused by medications impairing L-T4 absorption. This should be taken into account by future guidelines on hypothyroidism management.

## Highlights

• L-T4 malabsorption is frequently encountered in clinical practice.
• Drug induced L-T4 malabsorption has metabolic and cardiovascular consequences.
• Control of TSH is not enough when drug induced L-T4 malabsorption is suspected.

## Introduction

Oral levothyroxine (L-T4) is the recommended hormone therapy for hypothyroidism, a therapy that is monitored by periodic measurements of serum TSH levels [1], [2] when the hypothyroidism etiology is primary. Undertreated primary hypothyroidism, that is elevation of serum TSH above target levels, is observed in approximately 20% of primary hypothyroid patients treated with tablet L-T4 [3], [4]. In approximately one-third of such patients, the cause of undertreatment is oral ingestion of one or more medications that interfere with the intestinal absorption of L-T4 [3]. Several lists of such interfering drugs are available (see Patients and Methods). The recommended serum TSH target level is ≤4.12 mU/L [1], [2], [3], which is the upper normal limit recorded by the Third National Health and Nutrition Examination Survey (NHANES III) [5]. However, for the National Academy of Clinical Biochemistry [6], which included some thyroid experts, and for other thyroid specialists (for instance, Ref. [7]), the upper normal limit of TSH should be 2.50 mU/L.

The issue of consequences from undertreated hypothyroidism is typically viewed in terms of persistent or partially corrected hypothyroidism symptomatology being associated with poor quality of life [8]. The 2014 ATA guidelines [2] devote one recommendation to the issue [“*4d. What are the potential deleterious effects of inadequate levothyroxine?*”] upon discussing the literature in 30 lines of text. The strong, moderate quality evidence recommendation is “*The adverse effects of thyroid hormone deficiency include detrimental effects on the serum lipid profile and progression of cardiovascular disease. We recommend that patients with overt hypothyroidism be treated with doses of levothyroxine that are adequate to normalize serum thyrotropin levels, in order to reduce or eliminate these undesirable effects*” [3], [4]. However, serum TSH levels in the upper part or above the upper limit of the reference range are associated with greater rates of metabolic disorders and cardiovascular disease, because of increased levels of fasting blood glucose, total cholesterol, LDL cholesterol, triglycerides, increased frequency of metabolic syndrome or type 2 diabetes mellitus [9], [10], [11], [12], [13], [14], [15], [16], [17].

Based on the above, in L-T4 treated primary hypothyroid patients who were coingesting tablet L-T4 and at least one interfering medicine, we aimed to assess the magnitude of increase in serum TSH and the occurrence of complications (namely, aggravation of preexisting or *de novo* appearance of metabolic and cardiovascular events).

## Material and methods

This is a retrospective study based on the analysis of computerized records of persons under the care of 8 family physicians; these physicians were previously involved in research studies [18], [19], [20]. The coauthors of this paper other than the 8 family physicians were blind to the identity of the persons.

### Patients

**Fig. 1:**
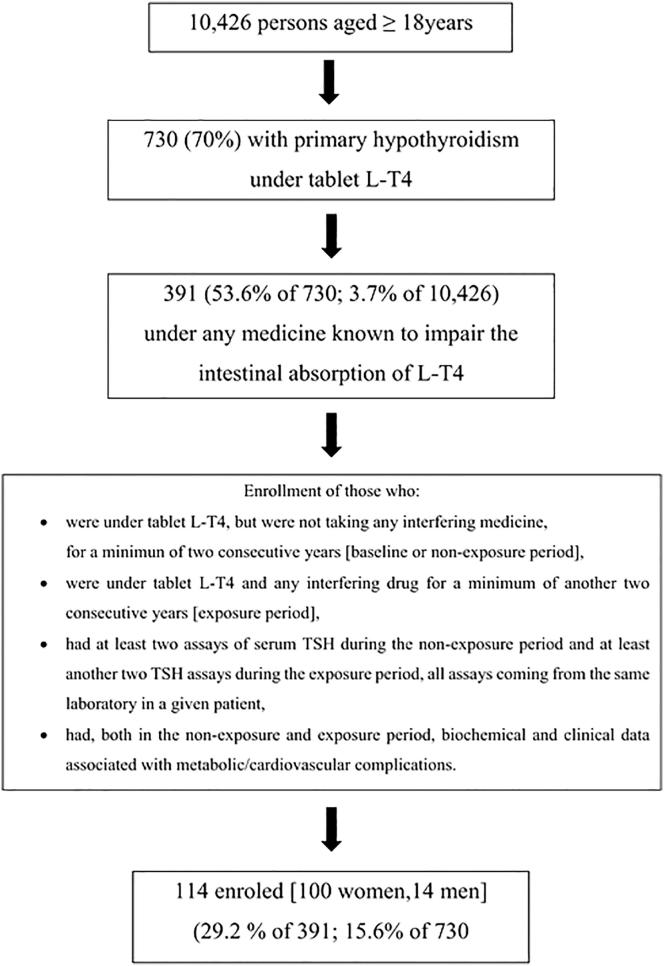
Flow chart of the study.

The flow-chart of the study is illustrated in Fig. 1.

First, in the 10,426 persons aged ≥18 years under the care of the 8 family physicians, we searched for those who were taking tablet L-T4 therapy, and retrieved 730 persons. The fundamental reasons for L-T4 therapy were autoimmune hypothyroidism and iatrogenic hypothyroidism (post-thyroidectomy almost always).

Second, we searched for persons who had taken one or more drugs known to inhibit the L-T4 intestinal absorption. We retrieved 391 such patients. The medications searched [1], [4], [21], [22], with the Anatomical Therapeutic Chemical (ATC) code given in parentheses, were: proton pump inhibitors (A02BC^*^), sucralfate (A02BX02), combination of aluminium hydroxide and magnesium hydroxide (A02AD01), calcium carbonate (A12AA04), calcium acetate (A12AA12), ferrous sulphate (B03AA07), raloxifene (G03XC01), cholestyramine (C10AC01), colestipol (C10AC02), lanthanum carbonate (V03AE03), cation exchange resins (V04CG01), and sevelamer (V03AE02).

Third, among these 391 patients we searched those who fulfilled our enrolment criteria for forming the final cohort, and retrieved 114 patients (29.1% of 391, and 15.6% of 730). These patients consisted of 100 women (88%) and 14 men (12%), and their mean age was 65.6 ± 12.7 years. Criteria for enrolment were: (i.) uninterrupted L-T4 therapy with tablet L-T4; (ii.) taking none of the said medications for at least 24 consecutive months in order to provide data for the period of time that we will refer to as “OFF interfering drug(s)” or non-exposure or baseline; (iii) taking any of the said medications for at least another 24 consecutive months in order to provide data for the period of time that we will refer to as “ON interfering drug(s)” or exposure; (iv.) having at least two non-exposure and at least two exposure determinations of serum TSH, all coming from the same laboratory in a given patient; (v.) having clinical and pertinent biochemical data associated with complications (see below) that were recorded within a period of at least two years during which serum TSH had remained on target while OFF interfering drug(s) and within a minimum of another two years while ON interfering drug(s). This criterion was important to compare the proportions of complications (exposure *versus* non-exposure to interfering drugs).

### Duration of the non-exposure and the exposure periods, and definition of complications

The databases of all 8 physicians record the date and number of prescriptions of any medicines, thus permitting to calculate the duration of treatment. The first day of ingestion of L-T4 or any interfering drug was the day after the date of the prescriptions. The last day of ingestion of L-T4 or the interfering drug(s) was either explicitly notated (for instance, because of adverse events) or deduced knowing the number of tablets/capsules in each prescribed package and the daily dose.

Concerning complications measurable biochemically, a precaution was taken not to compare the same biochemical index at very different ages. Once we determined the duration of the exposure period for each of the 114 patients, we opted to use a chronological window of similar duration to have the equivalent data for the non-exposure period (baseline).

In the 114 patients, the duration of the non-exposure period and exposure period was 35.4 ± 9.7 months (median 32.5; range 24–66) and 32.1 ± 6.9 months (median 31; range 24–55), respectively. The corresponding number of TSH determinations was 295 (2.6 per patient) and 335 (2.9 per patient).

Complications considered were *de novo* appearance or aggravation of any of the conditions listed and defined in Table 1 [23], [24], [25], [26], [27], [28].

**Table 1:** List of clinical/biochemical parameters whose *de novo* appearance or worsening (if pre-existing) was considered as complication.*

Parameter/Event	Definition	Definition of worsening
Metabolic syndrome	Based on NCEP ATP III classification, any three of five components: (i) abdominal obesity [AO], (ii) hypertension, (ii) (iii) hyperglycemia (impaired fasting glycemia [IFG] or diabetes mellitus [DM]), (iv) hypertriglyceridemia, (v) decreased HDL.	Progression to four or all five components, or aggravation of pre-existing component: (i) Abdominal obesity- further increase of waist circumference by >5%. (ii) Hypertension- see below, (hypertension) (iii) IFG- progression to DM (iv) DM- see below, (diabetes mellitus) (v) Hypertriglyceridemia- see below, (dyslipidemia) (vi) (v) Decreased HDL- further decrease by >10%
Dyslipidemia (any of hypercholesterolemia, hypertriglyceridemia, decreased HDL-cholesterol)	Hypercholesterolemia is total cholesterol ≥ 200 mg/dl.Hypertriglyceridemia is triglycerides ≥ 150 mg/dl or being on drug treatment for elevated triglycerides.Decreased HDL-cholesterol (HDL-C) is HDL-C ≤ 40 mg/dl in men and ≤ 50 mg/dl in women or being on drug treatment for reduced HDL-C.	For hypercholesterolemia and hypertriglyceridemia, any of: failure to reach target levels; increased posology of hypolipidemic drugs; addition of another hypolipidemic drug (typically, ezetimibe to a statin). For patients who refused lipid-lowering therapy, aggravation was defined as an increase in total cholesterol or triglycerides greater than 10%.For decreased HDL-cholesterol, see above (metabolic syndrome)
Impaired fasting glycemia (IFG)	Fasting blood glucose between 100 and 125 mg/dl	See metabolic syndrome
Diabetes mellitus	Fasting plasma blood glucose level of 126 mg/dl or greater on two separate occasions, or casual blood glucose taken at any time (including the second hr of the oral glucose tolerance test [OGTT]) that is ≥200 mg/dl.	Any of: HbA1c ≥ 7% (but ≥ 8% in frail old patients); appearance of one or more diabetes complications; aggravation of pre-existing complications (for instance, progression from microalbuminuria to macroalbuminuria; progression in retinopathy stages); hospitalization for diabetes-related problems; addition of one or more oral hypoglycemic agents; addition of insulin; increased daily doses of insulin
Hypertension	ESH/ESC Guidelines Classification into 6 categories (see footnote).	Any of: progression to a higher category; failure to reach target values of <140/90 mmHg; increased posology of a drug; addition of another antihypertensive drug; occurrence of any complication (heart, peripheral arteries, kidney, brain, retina).
Coronary heart disease (CHD)	Angina pectoris (AP): ICD-9-CM Diagnosis Code 413.9 Myocardial infarction (MI) ICD-9-CM Diagnosis Code 410 (410.0 through 410.9)	AP- Any of: subsequent AP, subsequent MI, congestive heart failure MI- Any of: subsequent MI, heart failure, mitral valve dysfunction, ventricular aneurism
Cerebrovascular disease (CVD)	Transient ischemic attack (TIA): ICD-CM Diagnosis Code 435 (435.0 through 435.9) Ischemic stroke (IS): ICD-CM Diagnosis Code 434 (434.01, 434.11, 434.91)	TIA- Any of: subsequent TIA or IS IS- Subsequent IS

Footnotes for Table 1.

Hypercholesterolemia is total cholesterol level ≥200 mg/dl (≥3.36 mmol/L), that is above the <200 mg/dl (<3.36 mmol/L) threshold considered by the NCEP ATP III to be desiderable.

According to the ESH/ESC Guidelines [25], blood pressure is classified into these categories, the category being defined by the highest level of pressure, whether systolic or diastolic. Categories were optimal (systolic < 120, diastolic < 80 mmHg), normal (systolic 120–129 and/or diastolic 81–84 mmHg), high-normal (systolic 130–139 and/or diastolic 85–89 mmHg), grade 1 hypertension (systolic 140–159 and/or diastolic 90–99 mmHg), grade 2 hypertension (systolic 160–179 and/or diastolic 100–109 mmHg), grade 3 hypertension (systolic ≥ 180 mmHg and/or diastolic ≥ 110 mmHg).

Definitions of worsening took into account Refs. [23], [24], [25], [26], [27], [28].

* Definitions for components of the metabolic syndrome [23]. (i) **Abdominal obesity**: waist circumference > 102 cm in men and > 88 cm in women. (ii) **Hypertension** = elevated systolic blood pressure ≥ 130 mm Hg or elevated diastolic blood pressure ≥ 85 mm Hg or being on antihypertensive drug treatment in a patient with a history of hypertension. (iii) **Hyperglycemia**: elevated fasting glucose ≥ 100 mg/dL or on drug treatment for elevated glucose. Precisely, as per the American Diabetes Association recommendation [24], these definitions are provided for impaired fasting glycemia (IFG), impaired glucose tolerance (IGT) and diabetes. IFG is fasting blood glucose between 100 and 125 mg/dl, and IGT is blood glucose between 140 and 199 mg/dl at the second hour of the OGTT. Diabetes is fasting plasma blood glucose level of 126 mg/dl or greater on two separate occasions, or casual blood glucose taken at any time (including the second hr of the OGTT) that is ≥ 200 mg/dl. (iv) **Hypertrigyceridemia**: elevated triglycerides ≥ 150 mg/dL (≥1.7 mmol/L) or being on drug treatment for elevated triglycerides. (v) **Reduced HDL-cholesterol**: HDL-C ≥ 40 mg/dL (≥1.03 mmol/L) in men and ≥ 50 mg/dL (≥1.3 mmol/L) in women or being on drug treatment for reduced HDL-C.

### Outcomes

These were continuous variables and categorical variables. The first consisted of mean ± SD of serum TSH. The second consisted of proportions of serum TSH above two different threshold levels (4.12 or 2.50 mU/L). Another categorical variable was proportions of *de novo* appearance or aggravation of any of the abnormalities (complications) mentioned above.

### Statistics

Differences between continuous variables were analysed by log_10_-transformed ANOVA, due to nongaussian distribution of serum TSH. Differences between categorical variables by the χ^2^ test or by Fisher’s exact test, as appropriate. Regardless of test, P values <0.05 were considered statistically significant.

## Results

### Medications interfering with L-T4 intestinal absorption

The spectrum of such medications in the 114 patients is summarized in Table 2. Proton pump inhibitors (PPI) were the medications most frequently used (95/114 [83%]), either alone (n = 71 [62%]) or in association with other interfering drugs (n = 24 [21%]). Calcium salts ranked the second, in that they were taken by 18 patients (16%) either alone or combined with other interfering drugs.

**Table 2:** Details of the medications known to interfere with the intestinal absorption of L-T4 that were taken by 114 patients with primary hypothyroidism under tablet L-T4 replacement therapy**.**

Drugs	No. of patients taking the drug *	Percentage §
Any	114	100
Calcium salts alone	5	4.4
Iron salts alone	2	1.7
Calcium + iron salts	4	3.5
Nonabsorbable antacids (NAA) alone	5	4.4
Ranitidine alone	3	2.6
Proton pump inhibitor (PPI) alone	71	62.3
PPI + NAA	12	10.5
PPI + calcium salt	5	4.4
PPI + iron salt	2	1.7
PPI + calcium salt + iron salt	1	0.9
PPI + NAA + calcium salt	3	2.6
PPI + NAA + iron salt	1	0.9

* Calcium salt was always calcium carbonate. Iron salt was always ferrous sulfate.

§ Denominator is always 114.

### First outcome: serum TSH levels during exposure *vs* non-exposure (baseline)

This comparison is shown in Table 3, where TSH is considered both as a continuous variable and a categorical variable based on two thresholds (see Introduction, and Patients and Methods).

**Table 3:** Serum TSH and proportions of TSH levels above two threshold levels (4.12 or 2.50 mU/L) in 114 patients with primary hypothyroidism who are stratified based on two periods of time, namely when they took L-T4 alone (OFF or non-exposure) and when they added ingestion of one or more drugs that interferes with the intestinal absorption of L-T4 (ON or exposure).

	Interfering drug		Statistics
	Off	On	On *vs* Off
No. of patients	114	114	
Serum TSH (mU/L), median	0.93	1.79 (+92%)	
mean	1.27	2.81 (+121%)	**P = 2.2 × 10^−20^**
SD	±1.34	±3.62	

No. of TSH measurements	295	335	
>4.12 mU/L	14 (4.7%)	62 (18.5%)	χ^2^ = 2.0, **P = 1.2 × 10-^7^** OR = 4.6 (95% CI: 2.5 to 8.3)
>2.50 mU/L	30 (10.2%)	96 (28.6%)	χ ^2^ = 33.5, **P = 7.1 × 10^−9^** OR = 3.5 (95% CI: 2.3 to 5.3)

No. of patients with at least one TSH measurement that was			
>4.12 mU/L	11 (9.6%)	41 (36.0%)	χ ^2^ = 22.4, **P = 2.2 × 10^-6^**OR = 5.3 (95% CI: 2.5 to 10.9)
>2.50/L	23 (20.2%)	61 (53.5%)	χ ^2^ = 27.2, P = **1.8 × 10^−7^** OR = 4.5 (95% CI: 2.2 to 8.2)

*The TSH thresholds (4.12 and 2.50 mU/L) considered are the one by the American thyroidologists [1] and the one by the National Academy of Clinical Biochemistry ([6].

Co-ingestion of tablet L-T4 and any interfering drug(s) was associated with serum TSH levels approximately 100% greater than those associated with ingestion of tablet L-T4 alone, the difference being extremely significant (P = 2.2 × 10^−20^).

### Second outcome: complications during exposure *vs* non-exposure (baseline)

Overall, aggravation of pre-existing relevant condition(s) or *de novo* appearance of it/them occurred in two-thirds of patients (76/114) (Table 4). Mean levels of TSH were 3-fold greater during exposure compared to non-exposure (P = 3.1 × 10^−34^) in the 76 patients who experienced complications, while they were insignificantly greater in the 38 patients who did not. Proportions of TSH measurements above either threshold were consistently greater during exposure compared to non-exposure. Such proportions were significantly greater in either above TSH threshold category (>4.12 or >2.5 mU/L) (Table 4, footnote).

**Table 4:** Intra-group comparison of serum TSH levels while on or off the interfering drug(s), the group being defined based on presence or absence of complications.*

	Interfering drug(s)		
Patients (n = 76) with complications	Off	On	Statistics (On *vs* Off)
No. of TSH measurements	199	222	
Serum TSH (mU/L), median	0.86	2.10 (+144%)	
mean	1.18	3.44 (+191%)	P = **3.1 × 10^−34^**
SD	±1.44	±4.08	

No. of TSH measurements that were			
>4.12 mU/L	10/199 (5.0%)	49/222 (22.1%)	χ ^2^ = 25.3, P = **4.9 × 10^−7^** OR = 5.3 (95% CI: 2.6–10.9)
>2.50 mU/L	21/199 (10.6%)	77/222 (34.7%)	χ 2 = 34.2 , P = **4.9 × 10^−9^** OR = 4.5 (95% CI: 2.6–7.6)

Patients with at least one TSH measurement that was			
>4.12 mU/L	7/76 (9.2%)	29/76 (38.2%)	χ ^2^ = 17.6, P = **2.7 × 10^−5^** OR = 6.1 (95% CI: 2.5–15.0)
>2.50 mU/L	15/76 (19.7%)	45/76 (59.2%)	χ ^2^ = 24.8, P = **6.4 × 10^−7^** OR = 5.9 (95% CI: 2.8–12.2)

**Patients (n = 38) without complications**			
No. of TSH measurements	96	113	
Serum TSH (mU/L), median	0.97	1.28 (+31. 9%)	P = 0.83
mean	1.43	1.58 (+10.5%)	
SD	± 1.09	± 1.98	

No. of TSH measurements that were			
>4.12 mU/L	4/96 (4.2%)	13/113 (11.5%)	χ ^2^ = 3.7, P = ***0.053*** OR = 3.0 (95% CI: 0.9 to 9.5)
>2.50 mU/L	9/96 (9.4%)	19/113 (16.8%)	χ ^2^ = 2.5, P = 0.11 OR = 1.9 (95% CI: 0.9 to 4.6)

Patients with at least one TSH measurement that was			
>4.12 mU/L	4/38 (10.5%)	12/38 (31.6%)	χ ^2^ = 5.1, **P = 0.024** OR = 3.9 (95% CI: 1.1–13.6)
>2.50 mU/L	8/38 (21.0%)	16/38 (42.1%)	χ ^2^ = 3.9, **P = 0.048** OR = 2.7 (95% CI: 1.0–7.5)

Intergroup comparison (complication group *vs* the non-complication group) when OFF interfering drug(s). TSH: 1.18 ± 1.44 *vs* 1.43 ± 1.09 mU/L (**P = 0.0086**). TSH > 4.12 mU/L: 10/199 *vs* 4/96 (P = 0.74), 7/76 *vs* 4/38 (P = 0.82). TSH > 2.50 mU/L: 21/199 *vs* 9/96 (P = 0.75), 15/76 *vs* 8/38 (P = 0.87).

Intergroup comparison (complication group *vs* the non-complication group) when ON interfering drug(s). TSH: 3.44 ± 4.08 *vs* 1.58 ± 1.98 mU/L (**P = <0.001**). TSH > 4.12 mU/L: 49/222 *vs* 13/113 (χ ^2^ = 5.5, **P = 0.018**, OR = 2.2 [95% CI 1.1 to 4.2]), 29/76 *vs* 12/38 (P = 0.49). TSH > 2.50 mU/L: 77/222 *vs* 19/113 (χ ^2^ = 11.7, **P = 0.0006**, OR = 2.6 [95% CI 1.6 to 4.6]), 45/76 *vs* 16/38 (χ ^2^ = 2.98, ***P = 0.08***, OR = 2.0 [95% CI 0.9 to 4.4]).

* P values typed **boldface** are statistically significant, while P values typed ***boldface italics*** are borderline statistically significant (P between 0.10 and 0.05).

### Relationship of complications with number of TSH measurements above thresholds

Based on a 3-tier categorization (one, two or ≥three TSH measurements above 4.12 mU/L during exposure), the 29 patients in the complication group were distributed significantly differently from the 12 patients in the noncomplication group (P = 0.007) (Table 5). Finally, in the 86 measurements from the 29 complication patients, serum TSH was significantly greater than in the 41 measurements from the 12 noncomplication patients (5.88 ± 5.68 mU/L [median 4.66] *vs* 2.54 ± 2.98 [median 0.96], P = 2.9 × 10^−6^) (Table 5, footnote). The whole pattern held upon using the threshold of 2.5 mU/L (Table 5).

**Table 5:** Patients with at least one TSH level above the specified threshold during the ON interfering drug period who are stratified based on the occurrence or non occurrence of complications during the same period.*

TSH threshold	Patients with complications	Patients without complications	Statistics
Measurements > 4.12 mU/L §	49/86 (57.0%)	13/41 (31.7%)	Df = 1, χ^2^ = 7.1, **P = 0.007**, OR = 2.8 [95% CI 1.3 to 6.2]
No. of patients	29	12	

No. of TSH measurements > 4.12 mU/L			
One	11/29 (37.9%)	11/12 (91.7%)	Df = 2, χ^2^ = 9.89, **P = 0.007**
Two	16/29 (55.2%)	1/12 (8.3%)	
≥Three	2/29 (6.9%)	0/12	

Measurements > 2.50 mU/L ^	77/130 (59.2%)	19/49 (38.8%)	Df = 1, χ^2^ = 5.99, **P = 0.014**, OR = 2.3 [95% CI 1.2 to 4.5]
No. of patients	45	16	

No. of TSH measurements > 2.50 mU/L			
One	19/45 (42.2%)	13/16 (81.2%)	Df = 2, χ2 = 7.63, **P = 0.022**
Two	20/45 (44.4%)	3/16 (18.8%)	
≥Three	6/45 (13.4%)	0/16	

* For definition of complications, see Table 1 and related footnotes. For details of complications in the 2 patients who had at least one TSH assay above the higher threshold (4.12 mU/L), see Table 5.

§ In the 86 measurements from the 29 patients with complications, TSH averaged 5.88 ± 5.68 mU/L (median 4.66), which is significantly greater than in the 41 measurements from the 12 patients without complications (2.54 ± 2.98 [median 0.96], P = 2.9 × 10^−6^).

^ In the 130 measurements from the 45 patients with complications, TSH averaged 4.75 ± 4.92 mU/L (median 3.12), which is significantly greater than in the 49 measurements from the 16 patients without complications (2.43 ± 2.77 [median 0.77], P = 3.2 × 10^−6^).

Noteworthy, 5/114 patients had no pre-existing abnormality during non-exposure and none appeared *de novo* during exposure. The corresponding TSH levels were 0.85 ± 0.43 (median 0.91) and 1.15 ± 0.59 mU/L (median 1.30), with none of a total of 31 TSH measurements being greater than 2.0 mU/L (data not shown).

### Relationship of number of complications with TSH measurements above thresholds

**Fig. 2:**
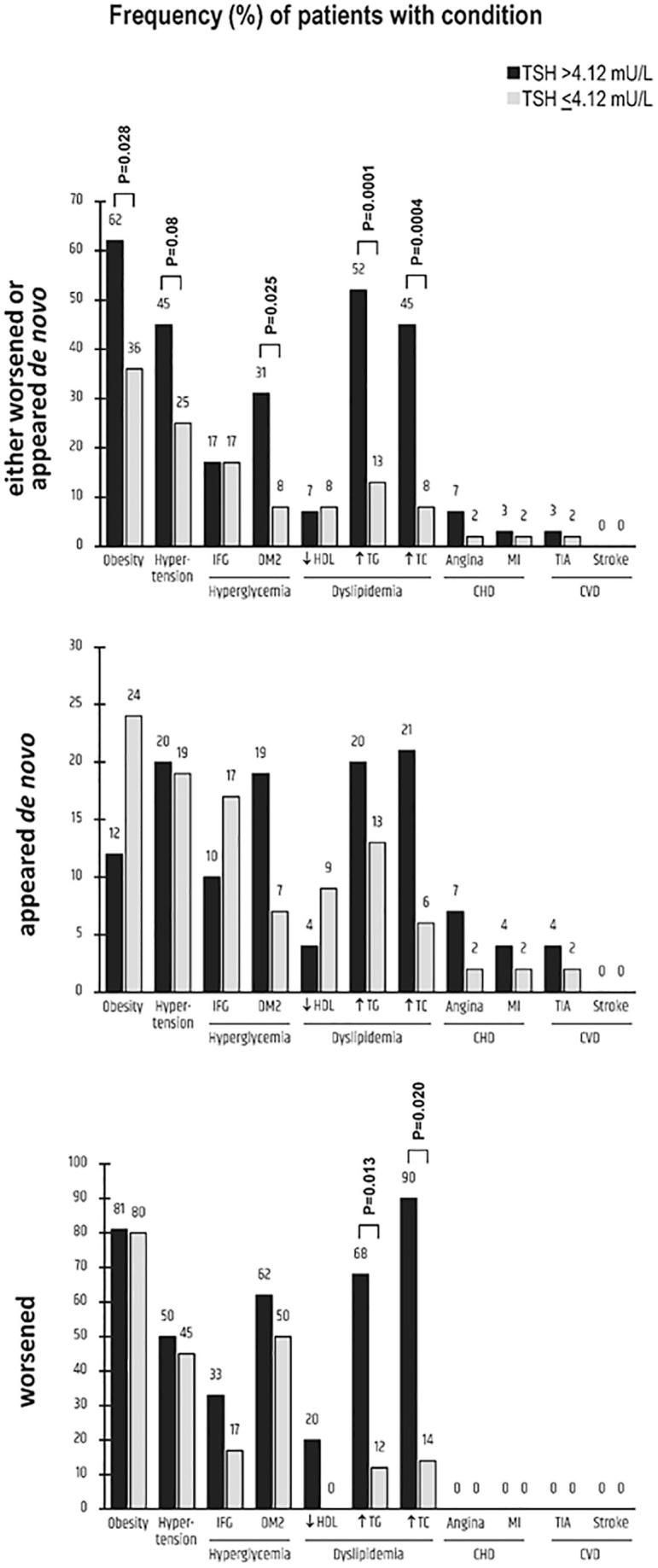
Details on clinical/biochemical conditions considered as complications in the group of the 76 patients in whom such conditions worsened (if pre-existing) or appeared *de novo* when exposed to drugs impairing the intestinal absorption of L-T4. Patients were stratified based on serum TSH levels (on target [≤4.12 mUL] or not on target [>4.12 mU/L]). For further details on the numbers appearing in the figure, see Table 6.

**Fig. 3:**
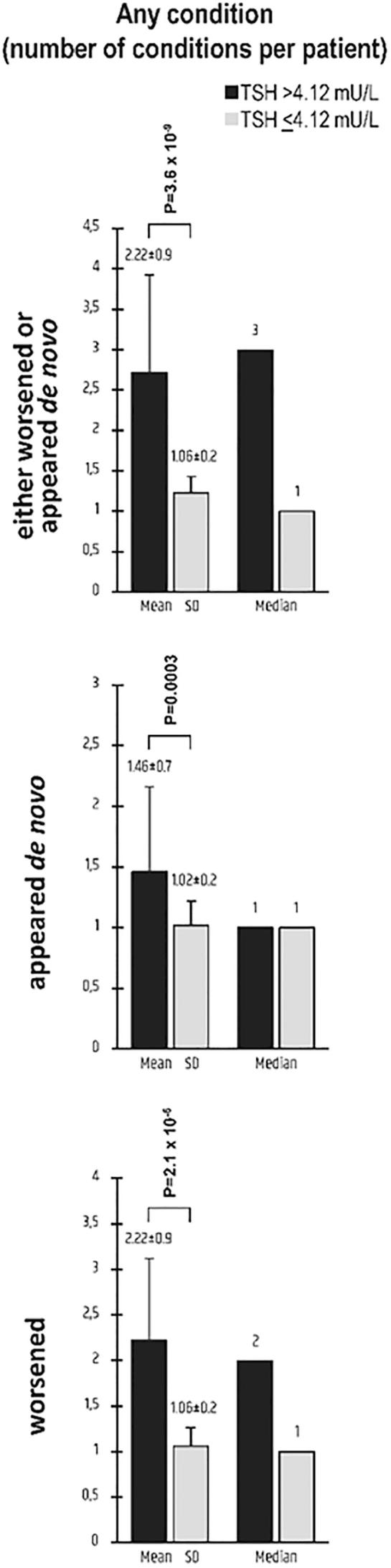
Number of conditions per patient that were considered as complications, either worsened (if pre-existing) or appeared *de novo*, upon stratifying patients based on serum TSH levels (on target [≤4.12 mUL] or not on target [>4.12 mU/L]). Graphically illustrated here are data reported in the last raw of Table.

Details on each complication in the whole group of the 76 patients and the two subgroups resulting from the target TSH-based stratification (4.12 mU/L threshold) are shown in Table 6. The two subgroups are graphically contrasted in Fig. 2, Fig. 3. By definition, during exposure no preexisting condition worsened or appeared *de novo* (if not pre-existing) in the remaining 38 patients (Fig. 2).

**Table 6:** Details on the conditions that were considered “complications” if pre-existing (in the OFF-interfering drug period) and worsening (in the ON-interfering drug period) or not pre-existing (in the OFF-interfering drug period) and appearing *de novo* (in the ON-interfering drug period).*

Condition	All (n = 76)			TSH > 4.12 mU/L (n = 29)			TSH ≤ 4.12 mU/L (n = 47)		
	Worsening	*De novo*	Either	Worsening	*De novo*	Either	Worsening	*De novo*	Either
Obesity	25/31 (80.6%)	10/45 (22.2%)	35/76 (46%)	17/21 (81%)	1/8 (12.5%)	**18/29 (62.1%)**	8/10 (80%)	9/37 (24.3%)	**17/47 (36.2%)**
Hypertension	17/35 (48.6%)	8/41 (19.5%)	25/76 (32.9%)	12/24 (50%)	1/5 (20%)	***13/29 (44.8%)***	5/11 (45.4%)	7/36 (19.4%)	***12/47 (25.5%)***
Hyperglycemia IFG	4/15 (26.7%)	9/61 (14.7%)	13/76 (17.1%)	3/9 (33.3%)	2/20 (10%)	5/29 (17.2%)	1/6 (16.7%)	7/41 (17.1%)	8/47 (17%)
Diabetes	6/10 (60%)	7/66 (10.6%)	13/76 (17.1%)	5/8 (62.5%)	4/21 (19%)	**9/29 (31%)**	½ (50%)	3/45 (6.7%)	**4/47 (8.5%)**
Reduced HDL	1/9 (11.1%)	5/67 (7.5%)	6/76 (7.9%)	1/5 (20%)	1/24 (4.2%)	2/29 (6.9%)	0/4	4/43 (9.3%)	4/47 (8.5%)
HyperTG	14/27 (51.8%)	7/49 (14.3%)	21/76 (27.6%)	**13/19 (68.4%)**	2/20 (20%)	**15/29 (51.7%)**	**1/8 (12.5%)**	5/39 (12.8%)	**6/47 (12.8%)**
Hypercholest	11/24 (45.8%)	6/52 (11.5%)	17/76 (22.4%)	**9/10 (90%)**	4/19 (21%)	**13/29 (44.8%)**	**2/14 (14.3%)**	2/33 (6.1%)	**4/47 (8.5%)**
CHD Angina	0/2	3/74 (4.0%)	3/76 (3.9%)	0/2	2/27 (7.4%)	2/29 (6.9%)	0/0	1/47 (2.1%)	1/47 (2.1%)
M infarction				0/1	1/28 (3.6%)	1/29 (3.4%)	0/0	1/47 (2.1%)	1/47 (2.1%)
CVD									
TIA	0/1	2/75 (2.7%)	2/76 (2.6%)	0/2	1/27 (3.7%)	1/29 (3.4%)	0/0	1/47 (2.1%)	1/47 (2.1%)
Stroke	0/76	0/76	0/76	0/29	0/29	0/29	0/0	0/29	0/29
Any of the aboveper pt [median] m ± SD	78 [1] 1.77 ± 0.9	59 [1] 1.13 ± 0.4	137 [1] 1.80 ± 1.1	60 [2] **2.22 ± 0.9**	19 [1] **1.46 ± 0.7**	79 [3]**2.72 ± 1.2**	18[1] **1.06 ± 0.2**	40 [1] **1.02 ± 0.2**	58 [1] **1.23 ± 0.5**

* Stratification in the two groups based on TSH levels in the ON-interfering period (TSH > 4.12 mU/L or ≤ 4.12 mU/L) indicates that the first group had at least one TSH measurement was above 4.12 mU/L, while the second group had all of the TSH measurements equal to or below 4.12 mU/L.

In the comparison of the 29 patients *vs* the 47 patients, the proportion of the following conditions were statistically different or borderline different for worsening of a pre-existing condition: hypertriglyceridemia (13/19 *vs* 1/8, **P = 0.013** by Fisher’s exact test, OR = 15.2 [95% CI 1.5–152]) and hypercholesterolemia (9/10 *vs* 2/14, **P = 0.0005**, OR = 54 [4.2 to 693]). The following proportions for either worsening of pre-existing condition or *de novo* appearance of a condition were significantly different or borderline different: Obesity (18/29 *vs* 17/47, χ^2^ = 4.8, **P = 0.028**, OR = 2.9 [95% CI 1.1 to 7.5]); hypertension (13/29 *vs* 12/47, χ^2^ = 3.0, ***P = 0.08***, OR = 1.7 [0.9 to 6.3]); Diabetes (9/29 *vs* 4/47, **P = 0.025** by Fisher’s exact test, OR = 4.8 [1.3 to 17.6]); hypertriglyceridemia (15/29 *vs* 6/47, χ^2^ = 13.6, **P = 0.0001**, OR = 7.3 [2.4 to 22.6]); hypercholesterolemia (13/29 *vs* 4/47, **P = 0.0004** by Fisher’s exact test, OR = 8.7 [2.5 to 30.8]). Proportions significantly different are typed boldface, while proportions borderline different ate typed boldface italics.

In the comparison between the 29 patients with the 47 patients, m ± SD of the following conditions were statistically different or borderline different: 2.22 ± 0.9 *vs* 1.06 ± 0.2 complications per patient (**P = 2.1x10^-5^**); 1.46 ± 0.7 *vs* 1.02 ± 0.2 (**P = 0.00032**); 2.72 ± 1.2 *vs* 1.23 ± 0.5 (**P = 3.6x10^-9^**).

When considering the pre-existing conditions (at least one of which worsened or appeared *de novo* in the group of the 76 patients), they occurred with statistically similar frequency in the 76 patients and the remaining 38 patients [not shown]. The exceptions were the significant difference concerning abdominal obesity (31/76 [40.8%] *vs* 7/38 [18.4%], χ^2^ = 5.7, P = 0.017, OR = 3.05 [95% CI 1.2–7.8]) and the borderline significant difference concerning reduced HDL (9/76 [11.8%] *vs* 10/38 [26.3%], χ^2^ = 3.8, P = 0.051, OR = 0.38 [95% CI 0.14–1.0]) [not shown]. The proportions of patients having at least one pre-existing condition were also borderline significantly different (54/76 [71.1%] *vs* 33/38 [86.8%], χ^2^ = 3.49, P = 0.061, OR = 0.38 [95% CI 0.1–1.1]) [not shown].

In the 76 patients there were 137 complications (mean = 1.80 per patient), with 48 patients having experienced a total of 78 aggravations of at least one preexisting condition (mean = 1.77 per patient) and 52 having experienced the *de novo* appearance of a total of 59 abnormal conditions (mean = 1.13 per patient). These numbers are significantly worse in the subgroup of patients (n = 29) with at least one TSH measurements >4.12 mU/L during exposure compared to the subgroup with none of such measurements above this threshold.

The conditions significantly linked to increased TSH levels appeared to be obesity, diabetes, hypertriglyceridemia and hypercholesterolemia (Table 6). Because of changes in these first three conditions, there were 3 new cases of metabolic syndrome in the subgroup of the 29 patients (25/29 compared to 22/29 at baseline). In contrast, within the subgroup of the remaining 47 patients the cases of metabolic syndrome remained 10. Furthermore, in these 22 patients, a total of 47 criteria (mean = 2.14 per patient) required for the NCEP ATPIII diagnosis of metabolic syndrome did worsen, which compares with 10 criteria in the 10 patients (mean = 1.0 per patient) of the subgroup with TSH ≤ 4.12 mU/L (data not shown in Table 6). Also, in the 38 patients who experienced no complications during exposure there were 10 with pre-existing metabolic syndrome. These 10 patients continued to have metabolic syndrome during exposure, but none of the NCEP ATPIII criteria worsened.

## Discussion

There are strengths in our work, which counterbalance what at first sight is the limitation of being retrospective. Indeed, one advantage of the retrospective nature of this study was lack of any bias, such as increasing the frequency of monitoring clinically/biochemically patients while on the interfering drugs. Second, the 8 physicians share a careful methodology in recording themselves clinical, laboratory, instrumental, pharmacological data of their patients, whom they knew very well. This is different from extracting data from a database that had been entered by an unknown number of different persons with an unknown methodology. Third, for both the unexposed period and the exposed period each patient has at least two determinations of TSH, and in a given patient this minimum of 4 TSH assays were performed in the same laboratory. Fourth, we have evaluated a number of complications that are sensitive to changes in serum TSH, not just one or two.

There are some major findings of our work. First, approximately one in two hypothyroid patients taking tablet L-T4 therapy (391/730) also takes one or more medications that interfere with L-T4 intestinal absorption. When these patients under polypharmacy are selected to have a minimum of two TSH measurements prior to and during polypharmacy (exposure), then around one-third of them (41/114) have at least one TSH measurement above 4.12 mU/L during exposure, a rate that is only one-tenth at baseline. The second finding has to do with “complications”, namely, the aggravation of pre-existing or the *de novo* appearance of any of the metabolic and cardiovascular conditions we have considered. Clearly, such complications are expected no matter what is the cause of elevated TSH. What we found, as a data previously unreported in the literature, is that those complication occur after a period of 24–55 months (median 31) of polytherapy in two-thirds of the tablet L-T4 replaced patients. During exposure, almost 40% of the patients with complications (29/76) had at least one TSH measurement >4.12 mU/L compared with approximately 10% (7/76) at baseline. Third, the link between undertreated hypothyroidism (TSH > 4.12 mU/L) and complications is underscored by a sort of “carry-over” effect. Indeed, 24% (7/29) of the patients with complications who had at least one TSH measurement >4.12 mUL during exposure also had at least one such measurement at baseline, which contrasts with the frequency of 8% (1/12) in the noncomplication group. Fourth, the said link is reinforced by the greater rate of complications in the subgroup of patients having at least one TSH measurement >4.12 mU/L during exposure compared with the subgroup having all TSH measurements ≤4.12 mU/L during exposure (Table 5). Fifth, data are congruent by looking from the opposite perspective, namely absence of complications. Indeed, there were few patients (n = 5, with a total of 31 TSH measurements) who had no alteration in the biochemical or clinical parameters both during exposure and at baseline. Of the 31 TSH measurements, none was greater than 2.0 mU/L.

When using the stringent threshold of 2.5 mU/L suggested by the National Academy of Clinical Biochemistry [6], the frequency of serum TSH levels above target continued to be significantly greater than in patients without complications (35% *vs* 17%, OR = 1.9), then one should conclude that a “desirable” level of serum TSH should be, indeed, below 2.5 mU/L.

Several studies have found significant unfavourable relationships between increases in serum TSH, even within the reference range, and parameters that we have considered to indicate complications [9], [10], [11], [12], [13], [14], [15], [16], [17]. For instance, the large studies from Norway have found progressively greater levels of total cholesterol, LDL-cholesterol, triglycerides [14], SBP and DBP [15], [16] along progressively greater bands of circulating TSH. In our study, it is noteworthy that the average TSH levels during exposure were 3.44 ± 4.08 mU/L (median 2.10) in the 76 patients who experienced complications but 1.58 ± 1.98 (median 1.28) in the 38 patients who did not. These two exposure average levels of TSH represent a great and a negligible increase compared to the corresponding baseline average levels (1.18 ± 1.44 mU/L [median 0.86] and 1.43 ± 1.09 [median 0.97]). In sum, the interference on the intestinal absorption of tablet L-T4 caused by certain medications, moves TSH from one class of metabolic/cardiovascular risk towards classes of greater risk.

Given the retrospective study design, association of elevated TSH and increased metabolic/cardiovascular risk could be due to a possible suboptimal compliance with medications in general that should be kept in mind. In addition, obesity and weight gain is associated with higher L-T4 requirements, thus a not fully uptitrated L-T4 dose could have led to increased TSH and metabolic/cardiovascular consequences [30].

Based on recommendation no. 13 of the 2012 ATA/AACE Guidelines, “*patients being treated for established hypothyroidism should have serum TSH measurements done at 4–8 weeks after initiating treatment or after a change in dose. Once an adequate replacement dose has been determined, periodic TSH measurements should be done after 6 months and then at 12-month intervals, or more frequently if the clinical situation dictates otherwise*” [1]. No recommendation on periodic TSH measurements are given by these [1] and subsequent guidelines [2] for hypothyroid patients who are under medications that increase thyroid hormone requirement. Based on the responses of almost 900 endocrinologists to a survey [29], after achieving stable target TSH levels, 55.5% of respondents recheck thyroid laboratory tests at 6-month intervals and 34% at 12-month intervals. Furthermore, once target TSH levels are achieved, approximately one-third of respondents (but half, if European) return the patient to the primary care physicians. In view of our data, hypothyroid patients under medications that affect thyroid hormone absorption/metabolism deserve more frequent rechecks.

## Conclusion

L-T4 treated hypothyroid patients who start taking any medications known to cause undertreatment of hypothyroidism should mention this adjunctive therapy to the physician who is managing their hypothyroidism. Periodic measurements of TSH cannot be sporadic but relatively frequent, and it should be complemented by appropriate evaluation of pre-existing or *de novo* appearance of metabolic and cardiovascular disorders. Future guidelines should enforce this more comprehensive strategy for following up this category of hypothyroid patients.

## Declarations of interest

None.

## References

[1] Garber J.R., Cobin R.H., Gharib H., Hennessey J.V., Klein I., Mechanick J.I. (2012). American association of clinical E, American thyroid association taskforce on hypothyroidism: clinical practice guidelines for hypothyroidism for hypothyroidism in adults: cosponsored by the American association of clinical endocrinologists and the American thyroid association. Thyroid.

[2] Jonklaas J., Bianco A.C., Bauer A.J., Burman K.D., Cappola A.R., Celi F.S. (2014). American Thyroid Association Task Force on Thyroid Hormone Replacement. Guidelines for the treatment of hypothyroidism: prepared by the American Thyroid Association task force on thyroid hormone replacement. Thyroid.

[3] Benvenga S. (2013). When thyroid hormone replacement is ineffective?. Curr Opin Endocrinol Diabetes Obes.

[4] Jonklaas J., Braverman L.E., Cooper D.S. (2013). Treatment of hypothyroidism. Werner and Ingbar’s the thyroid: a fundamental and clinical text.

[5] Hollowell J.G., Staehling N.W., Flanders W.D., Hannon W.H., Gunter E.W., Spencer C.A. (2002). Serum TSH, T(4), and thyroid antibodies in the United States population (1988 to 1994): National Health and Nutrition Examination Survey (NHANES III). J Clin Endocrinol Metab.

[6] Baloch Z., Carayon P., Conte-Devolx B., Demers L.M., Feldt-Rasmussen U., Henry J.F. (2003). Laboratory support for the diagnosis and monitoring of thyroid disease. Thyroid.

[7] Wartofsky L., Dickey R.A. (2005). The evidence for a narrower thyrotropin reference range is compelling. J Clin Endocrinol Metab.

[8] Vigário Pdos S., Vaisman F., Coeli C.M., Ward L., Graf H., Carvalho G. (2013). Inadequate levothyroxine replacement for primary hypothyroidism is associated with poor health-related quality of life-a Brazilian multicentre study. Endocrine.

[9] Park H.T., Cho G.J., Ahn K.H., Shin J.H., Hong S.C., Kim T. (2009). Thyroid stimulating hormone is associated with metabolic syndrome in euthyroid postmenopausal women. Maturitas.

[10] Laclaustra M., Hurtado-Roca Y., Sendin M., Leon M., Ledesma M., Andres E. (2015). Lower-normal TSH is associated with better metabolic risk factors: a cross-sectional study on Spanish men. Nutr Metab Cardiovasc Dis.

[11] Benseñor I.M., Goulart A.C., Molina Mdel C., de Miranda É.J., Santos I.S., Lotufo P.A. (2015). Thyrotropin levels, insulin resistance, and metabolic syndrome: a cross-sectional analysis in the Brazilian Longitudinal Study of Adult Health (ELSA-Brasil). Metab Syndr Relat Disord.

[12] Giandalia A., Russo G.T., Romeo E.L., Alibrandi A., Villari P., Mirto A.A. (2014). Influence of high-normal serum TSH levels on major cardiovascular risk factors and Visceral Adiposity Index in euthyroid type 2 diabetic subjects. Endocrine.

[13] Jun J.E., Jin S.M., Jee J.H., Bae J.C., Hur K.Y., Lee M.K. (2017). TSH increment and the risk of incident type 2 diabetes mellitus in euthyroid subjects. Endocrine.

[14] Asvold B.O., Vatten L.J., Nilsen T.I., Bjøro T. (2007). The association between TSH within the reference range and serum lipid concentrations in a population-based study. The HUNT Study. Eur J Endocrinol.

[15] Asvold B.O., Bjøro T., Nilsen T.I., Vatten L.J. (2007). Association between blood pressure and serum thyroid-stimulating hormone concentration within the reference range: a population-based study. J Clin Endocrinol Metab.

[16] Iqbal A., Figenschau Y., Jorde R. (2006). Blood pressure in relation to serum thyrotropin: the Tromsø study. J Hum Hypertens.

[17] Gumieniak O., Perlstein T.S., Hopkins P.N., Brown N.J., Murphey L.J., Jeunemaitre X. (2004). Thyroid function and blood pressure homeostasis in euthyroid subjects. J Clin Endocrinol Metab.

[18] Cacciola I., Scoglio R., Alibrandi A., Squadrito G., Raimondo G., SIMG-Messina Hypertransaminasemia Study Group (2017). Evaluation of liver enzyme levels and identification of asymptomatic liver disease patients in primary care. Intern Emerg Med..

[19] Alecci U., Bonina F., Bonina A., Rizza L., Inferrera S., Mannucci C. (2016). Efficacy and safety of a natural remedy for the treatment of gastroesophageal reflux: a double-blinded randomized-controlled study. Evidence Based Complement Alternat Med.

[20] Mazzaglia G., Arcoraci V., Cutroneo P., Inferrera S., Alecci U., Bonfiglio S. (1998). Infectious diseases in general practice and antibiotic prescription. Observational study in Sicily. Recenti Prog Med.

[21] Colucci P.H., Seng Yue C., Ducharme M., Benvenga S. (2013). A review of the pharmacokinetics of levothyroxine for the treatment of hypothyroidism. Eur Endocrinol.

[22] Virili C., Antonelli A., Santaguida M.G., Benvenga S., Centanni M. (2018). Gastrointestinal malabsorption of thyroxine. Endocr Rev.

[23] Grundy S.M., Cleeman J.I., Daniels S.R., Donato K.A., Eckel R.H., Franklin B.A. (2005). Diagnosis and management of the metabolic syndrome: an American Heart Association/National Heart, Lung, and Blood Institute Scientific Statement. Circulation.

[24] Genuth S., Alberti K.G., Bennett P., Buse J., Defronzo R., Kahn R. (2003). Expert committee on the diagnosis and classification of diabetes mellitus. Follow-up report on the diagnosis of diabetes mellitus. Diabetes Care.

[25] Mancia G., Fagard R., Narkiewicz K., Redón J., Zanchetti A., Böhm M. (2013). ESH/ESC Guidelines for the management of arterial hypertension: the Task Force for the management of arterial hypertension of the European Society of Hypertension (ESH) and of the European Society of Cardiology (ESC). J Hypertens.

[26] Perk J., DeBacker G., Gohlke H., Graham I., Reiner Z., Verschuren M. (2012). European Guidelines on cardiovascular disease prevention in clinical practice (version 2012): the Fifth Joint Task Force of the European Society of Cardiology and Other Societies on Cardiovascular Disease Prevention in Clinical Practice (constituted by representatives of nine societies and by invited experts). Eur Heart J.

[27] Associazione Medici Diabetologi (AMD) – Società Italiana di Diabetologia (SID) – Standard italiani per la cura del diabete mellito 2014. Available from: www.standarditaliani.it.

[28] Morini E., Catalano A., Lasco A., Morabito N., Benvenga S. (2018). L-thyroxine malabsorption due to calcium carbonate impairs blood pressure, total cholesterolemia, and fasting glycemia. Endocrine.

[29] Burch H.B., Burman K.D., Cooper D.S., Hennessey J.V. (2014). A 2013 survey of clinical practice patterns in the management of primary hypothyroidism. J Clin Endocrinol Metab.

[30] Glymph K., Gosmanov A.R. (2016). Levothyroxine replacement in obese hypothyroid females after total thyroidectomy. Endocr Pract.

